# Supplementary material for: Increasing leaf sizes of the vine Epipremnum aureum (Araceae): photosynthesis and respiration
Source: PeerJ. 2025 Apr 4;13:e19214. doi: 10.7717/peerj.19214 (PMC11974542; doi:10.7717/peerj.19214)
Supplement: Supplemental Information 2 — Statistical significance results of t-test and Mann-Whitney comparing photosynthesis, morpho-physiology and anatomy parameters of Epipremnum aureum leaves under different light conditions (low and high light) and growth directions (horizontal and vertical). Bold values represent p < 0.05 [file peerj-13-19214-s002.docx]

Supplementary material

**Table S1**. Statistical significance results of *t-test* and Mann-Whitney comparing photosynthesis, morpho-physiology and anatomy parameters of *Epipremnum aureum* leaves under different light conditions and growth directions. Bold values represent P < 0.05

|  | **Same growth direction;**  **different light conditions** | | **Same light conditions;**  **different growth directions** | |
| --- | --- | --- | --- | --- |
| ***Morpho-physiology traits*** | **LLhoriz x HLhoriz** | **LLclimb x HLclim** | **LLhoriz x LLclimb** | **HLhoriz x HLclimb** |
| Leaf area | t=-40.97; 8df. P=0.260 | **P=0.008** | t=-23.29; 8df. P=0.308 | t=-13.217; 8df. **P<0.001** |
| Succ | t=-1.935; 8df. P=0.089 | t=-2.100; 8df. P=0.069 | t=+4.732; 8df. **P=0.001** | t=+4.599; 8df. **P=0.002** |
| SLA | **P=0.008** | t=+12.673; 8df. **P<0.001** | P=0.69 | t=-0.061; 8df. P=0.949 |
| N (%) | t= 0.46; 8df.  P=0.65 | t= -0.794; P=0.45 | t= 0.25; 8df.  P=0.80 | t= 0.092; 8df.  P=0.92 |
| ***Photosynthetic traits*** | **LLhoriz x HLhoriz** | **LLclimb x HLclim** | **LLhoriz x LLclimb** | **HLhoriz x HLclimb** |
| ETR | t=-5.92; 8df. **P<0.001** | t=-5.92; 8df. **P<0.001** | t=-1.62; 8df. P<0.144 | t=+0.21; 8df.  P=0.83 |
| Amax | t=-1.89; 8df. P=0.094 | t=-1.24; 8df. P=0.249 | t=+1.859; 8df. P=0.100 | t=+1.78; 8df. P=0.272 |
| LSP | t=-1.18; 8df. P=0.269 | P=0.421 | t=+0.088; 8df. P=0.93 | t=+0.61; 8df. P=0.559 |
| RD | t=-4.21; 8df. **P=0.003** | **P=0.008** | P=1.000 | t=-2.76; 8df. **P=0.024** |
| ***Leaf anatomy*** | **LLhoriz x HLhoriz** | **LLclimb x HLclim** | **LLhoriz x LLclimb** | **HLhoriz x HLclimb** |
| Leaf | t=-3.663; 8df. **P=0.006** | t=-1.08; 8df. P=0.312 | t=-0.55; 8df. P=0.594 | t=+2.67; 8df. **P=0.028** |
| Mesophyll | t=-5.118; 8df. **P<0.001** | t=-4.15; 8df. **P=0.002** | t=-0.37; 8df. P=0.72 | t=+1.695; 8df. P=0.129 |
| Palisade/  Spongy ratio | t=-0.42; 8df. P=0.685 | P=0.111 | t=-1.38; 8df. P=0.205 | t=+0.48; 8df. P=0.646 |
| %area | t=+0.231; 8df. P=0.820 | t=+1.266; 8df. P=0.240 | t=-0.31 8df. P=0.441 | t=+0.068; 8df. P=0.949 |
| VD | t=-2.131; 8df. P=0.06 | t=-3.089; 8df. **P=0.015** | t=+1.82; 8df. P=0.105 | t=-0.843; 8df. P=0.424 |
| SD | t=-1.206; 8df. P=0.262 | t=-4.07; 8df. **P=0.002** | t=+0.272; 8df. P=0.790 | t=-3.501; 8df. **P=0.008** |
| ***Plant growth Biomass*** | **LLhoriz x HLhoriz** | **LLclimb x HLclim** | **LLhoriz x LLclimb** | **HLhoriz x HLclimb** |
| RGR | t=-4.195; 8df. **P=0.003** | **P = 0.011** | P = 0.129 | t=-2.772; 8df.  **P= 0.024** |
| NAR | t=-4.165; 8df. **P=0.003** | **P = 0.012** | t=2.639; 8df. P=0.030 | t=-1.9227; 8df. P=0.091 |
