# Supplementary material for: Increasing leaf sizes of the vine Epipremnum aureum (Araceae): photosynthesis and respiration
Source: PeerJ. 2025 Apr 4;13:e19214. doi: 10.7717/peerj.19214 (PMC11974542; doi:10.7717/peerj.19214)
Supplement: Supplemental Information 3 — (A) Dry mass increment; (B) Leaf area increase. Closed symbols denote lowlight treatments (LL-horiz and LL-climb). Open symbols denote high light treatments (HL-horiz and HL-climb). Circles represent horizontal treatments (LL-horiz and HL-horiz) while climbing treatments (LL-climb and HL-climb) are represented by triangles (n = 5; mean ±s.d.). [file peerj-13-19214-s003.pdf]

Supplemental Material 3

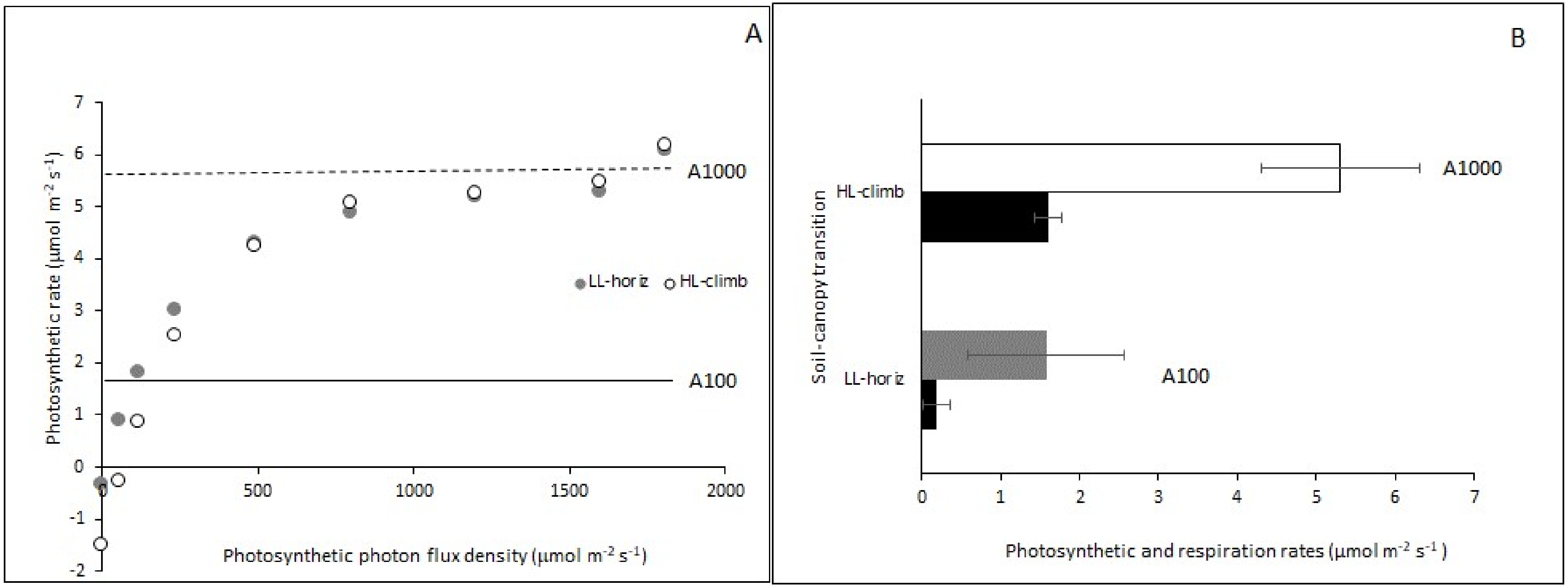

Figures A and B. Photosynthetic light rates for LL-horiz (gray circle) and HL-climb (white circle) leaves of *E. aureum* obtained via light curves (A) and applied for both treatments if occurring under natural light conditions (B). In figure A, mean values of leaf photosynthesis show similar light curves for both treatments. However, using the same curves, photosynthetic rate of smaller leaves at understory conditions (A100 line, gray bar) is much lower than at canopies (A1000 line, dashed line). Figure B depicts photosynthetic rate along soil (LL-horiz, A100) to canopy (HL-climb, A1000) transition using data from light curves. White bar indicates photosynthesis of large leaves (represented by HL-climb) at canopy conditions (A1000) and gray bar indicate photosynthesis of smaller leaves (represented by LL-horiz) at understory (A100). For the sake of comparison, the respective Rd value for each treatment is indicated (black bar).
