# Supplementary material for: Increasing leaf sizes of the vine Epipremnum aureum (Araceae): photosynthesis and respiration
Source: PeerJ. 2025 Apr 4;13:e19214. doi: 10.7717/peerj.19214 (PMC11974542; doi:10.7717/peerj.19214)

Supplementary material **Fig. S2. Plant growth of *Epipremnum aureum* growing under different light conditions (low and high light) and growth directions (horizontal and vertical) along 10 months of experiment.** (A) Dry mass increment; (B) Leaf area increase. Closed symbols denote lowlight treatments (LL-horiz and LL-climb). Open symbols denote high light treatments (HL-horiz and HL-climb). Circles represent horizontal treatments (LL-horiz and HL-horiz) while climbing treatments (LL-climb and HL-climb) are represented by triangles (n=5; mean ± s.d.).

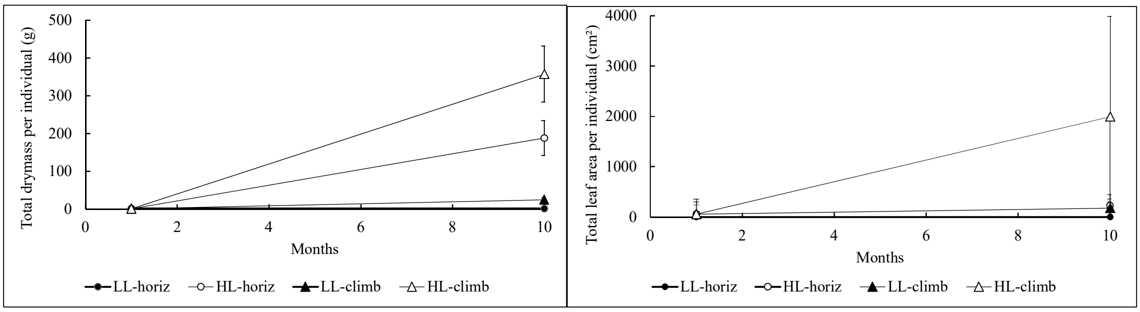

Supplement: Supplemental Information 4 — (A) Dry mass increment; (B) Leaf area increase. Closed symbols denote lowlight treatments (LL-horiz and LL-climb). Open symbols denote high light treatments (HL-horiz and HL-climb). Circles represent horizontal treatments (LL-horiz and HL-horiz) while climbing treatments (LL-climb and HL-climb) are represented by triangles (n = 5; mean ±s.d.). [file peerj-13-19214-s004.pdf]
