# Supplementary material for: Increasing leaf sizes of the vine Epipremnum aureum (Araceae): photosynthesis and respiration
Source: PeerJ. 2025 Apr 4;13:e19214. doi: 10.7717/peerj.19214 (PMC11974542; doi:10.7717/peerj.19214)

Fig. S3. Plant growth parameters of *E. aureum* leaves under different light conditions and growth directions. (A) Relative growth rate (RGR); (B) Net assimilation rate (NAR). Symbol * indicates statistical differences between different light conditions; symbol # indicates statistical differences between different growth directions ($p<0.05$; $n = 5$). LL-horiz: low light horizontal; HL-horiz: high light horizontal; LL-climb: low light vertical; and HL-climb: high light vertical.

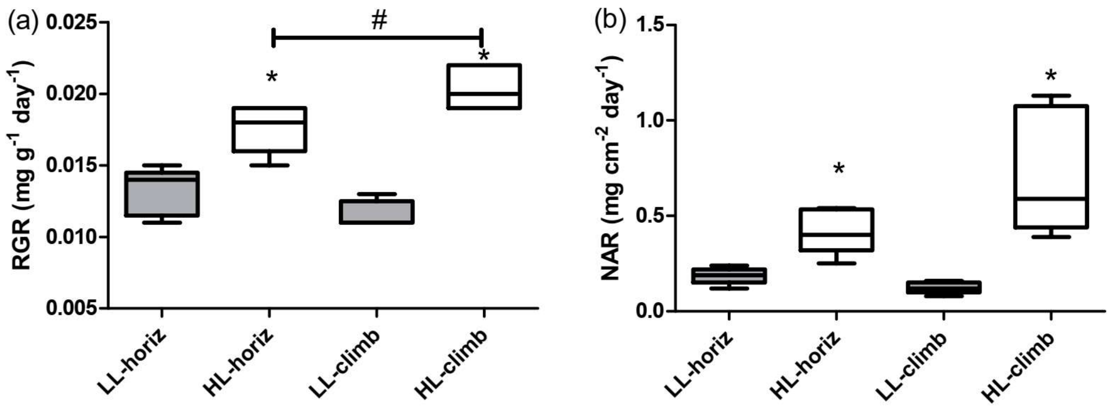

Supplement: Supplemental Information 5 — (A) Relative growth rate (RGR); (B) Net assimilation rate (NAR). Symbol * indicates statistical differences between different light conditions; symbol # indicates statistical differences between different growth directions (p < 0.05; n = 5). LL-horiz: low light horizontal; HL-horiz: high light horizontal; LL-climb: low light vertical; and HL-climb: high light vertical. [file peerj-13-19214-s005.pdf]
